# A Two-Minute Paper-and-Pencil Test of Symbolic and Nonsymbolic Numerical Magnitude Processing Explains Variability in Primary School Children's Arithmetic Competence

**DOI:** 10.1371/journal.pone.0067918

**Published:** 2013-07-02

**Authors:** Nadia Nosworthy, Stephanie Bugden, Lisa Archibald, Barrie Evans, Daniel Ansari

**Affiliations:** 1 Numerical Cognition Laboratory, Department of Psychology, University of Western Ontario, London, Ontario, Canada; 2 School of Communications Sciences and Disorders, Department of Psychology, University of Western Ontario, London, Ontario, Canada; 3 Psychological Services, Thames Valley District School Board, London, Ontario, Canada; University of Leicester, United Kingdom

## Abstract

Recently, there has been a growing emphasis on basic number processing competencies (such as the ability to judge which of two numbers is larger) and their role in predicting individual differences in school-relevant math achievement. Children’s ability to compare both symbolic (e.g. Arabic numerals) and nonsymbolic (e.g. dot arrays) magnitudes has been found to correlate with their math achievement. The available evidence, however, has focused on computerized paradigms, which may not always be suitable for universal, quick application in the classroom. Furthermore, it is currently unclear whether both symbolic and nonsymbolic magnitude comparison are related to children’s performance on tests of arithmetic competence and whether either of these factors relate to arithmetic achievement over and above other factors such as working memory and reading ability. In order to address these outstanding issues, we designed a quick (2 minute) paper-and-pencil tool to assess children’s ability to compare symbolic and nonsymbolic numerical magnitudes and assessed the degree to which performance on this measure explains individual differences in achievement. Children were required to cross out the larger of two, single-digit numerical magnitudes under time constraints. Results from a group of 160 children from grades 1–3 revealed that both symbolic and nonsymbolic number comparison accuracy were related to individual differences in arithmetic achievement. However, only symbolic number comparison performance accounted for unique variance in arithmetic achievement. The theoretical and practical implications of these findings are discussed which include the use of this measure as a possible tool for identifying students at risk for future difficulties in mathematics.

## Introduction

There is growing evidence to suggest math skills are just as important as reading skills when predicting a child’s academic success and competence in mathematics is crucial to one’s success in school and the workplace [1], [2]. Moreover, low numeracy skills are associated with worse health care, greater likelihood of criminal behaviour, as well as higher risk for depression and other illnesses [3].

Against this background, early identification of students at risk for developing poor math achievement should be a key priority of education systems and their teachers in the classroom. In the domain of reading, much progress in early diagnosis of at-risk children has been made by focusing on processing competencies that are foundational to reading, such as phonological awareness [4]–[6]. Currently, math skills are most frequently measured by using tests of skills that children are taught in school, such as basic calculation abilities. Such tests, however, do not necessarily tap into the foundational processes that allow children to acquire educationally-relevant skills, such as arithmetic fluency.

So what might be the foundational competencies that serve as a scaffold for children’s early mathematical learning? In order to process numbers it is necessary to have an understanding of the magnitudes they represent (e.g., knowing that the Arabic digit 3 stands for three items). Without an understanding of numerical magnitude and its association with numerical symbols the learning of mental arithmetic cannot get off the ground. Therefore, tests aiming to characterize the foundational skills of children’s numerical abilities should include measures of numerical magnitude processing. Research has shed light onto how numerical magnitudes are represented by adult humans [7]–[8] and over the last two decades, a large body of research has been amassed which demonstrates that even infants [9]–[11] and non-human species [12]–[14] are capable of numerical magnitude processing, when these magnitudes are represented nonsymbolically (e.g., arrays of dots). Evidence of numerical magnitude processing ability in infants and non-human animals and adults suggests that it is a basic, yet important skill in number processing and may provide the basis for learning the numerical meaning of numerical symbols.

To measure numerical magnitude processing in older children and adults, researchers have frequently employed number comparison paradigms in which participants are asked to choose which of two numbers is larger in numerical magnitude. When individuals compare numerical magnitudes, an inverse relationship between the numerical distance of two magnitudes and the reaction time required to make a correct comparison is obtained [7]. In other words, individuals are faster and more accurate at judging which of two numbers is numerically larger when the numbers are numerically more distant (e.g., 5 vs. 9) than when they are relatively close (e.g., 5 vs. 6). This relationship between numerical distance and response times and accuracy is known as the numerical distance effect (NDE). This effect has been found to change over developmental time [15]. Specifically, younger children exhibit relatively larger NDE’s compared to adolescents and adults who demonstrate comparatively smaller NDE’s.

To explain the numerical distance effect, one popular account posits that numerically close magnitudes have more representational features in common than those that are farther apart. Because of this, discriminating between a pair of numerical magnitudes is more challenging for quantities that are numerically closer together, which results in the NDE during comparison tasks [8]. A number of models have been put forth to explain the numerical distance effect and its underlying cognitive processes of numerical representation: the “accumulator” model [16], the “number line” model [8] and the “numerosity code” model [17]. Even though these models differ in their precise characterization of the underlying mental representations of numerical magnitude, they all concur that numerical magnitude comparison and the NDE provides an important metric of numerical magnitude processing.

Another effect that is observed in numerical magnitude comparison studies is the numerical ratio effect (NRE [7]). The NRE posits that individuals are faster and more accurate at comparing two numbers of a smaller magnitude versus two numbers of a larger magnitude, even when the distance between the numbers remains constant (i.e., 3, 4 vs. 8, 9, where it takes participants longer to judge that 9 is larger than 8 then it does them to decide that 4 is larger than 3). Both the NDE and the NRE can be observed with symbolic stimuli such as Arabic digits and nonsymbolic stimuli such as arrays of dots [18].

The finding that the numerical ratio between two numbers influences the speed with which they can be accurately compared is consistent with Weber’s Law which states that the just noticeable difference between two stimuli is directly proportional to the magnitude of the stimulus with which the comparison is being made. This is reflected in the NRE where a specific difference between two magnitudes results in a faster response time the smaller the absolute values of the magnitudes being compared.

Against the background of the review of the existing literature described above, it is clear that much has been uncovered about the characteristics of the representation and processing of both symbolic and nonsymbolic numerical magnitudes across development and species. A question resulting from this research, which has been a growing focus in recent years, is whether individual differences in basic number processing are related to between-subjects variability in mathematical achievement. In other words, are metrics of numerical magnitude processing, such as the numerical distance and numerical ratio effects, meaningful predictors of individual differences in children’s level of mathematical competence? And if so, can such measures be used to detect children at risk of developing mathematical learning difficulties, such as developmental dyscalculia?

In recent years, a growing number of studies have begun to answer this question. In one of the pioneering studies in this area, Durand, Hulme, Larkin and Snowling [19] studied typically developing children between the ages of 7–10 years. Participants’ ability to compare symbolic numerical magnitudes (Arabic digits) as rapidly and as accurately as possible was assessed using a numerical comparison task. In this task, participants were required to judge which of two digits was numerically larger. The digits used ranged from 3–9 and the numerical distance between pairs was either one or two. Participants had a 30 second time limit to complete 28 questions in which they responded by choosing the larger magnitude in each pair. In addition, children’s arithmetic skills were measured using the Numerical Operations subtest of the Wechsler Objective Numerical Dimensions (WOND). In the WOND children are required to write Arabic numerals and complete simple and multi-digit addition, subtraction, multiplication and division problems. Other items in the subtest involved fractions, decimals and negative numbers. Participants were also given an arithmetic task in which they had one minute to answer as many addition and subtraction problems as possible. The results of the study indicated that individual differences in the accuracy of symbolic numerical magnitude comparison were associated with between-subject variability in arithmetic ability: students with higher accuracy on the digit comparison task were better at solving addition and subtraction problems and received higher scores on the WOND than students who performed comparatively more poorly on the number comparison task. This finding demonstrates that a very basic skill such as magnitude comparison is related to children’s performance on higher order math skills.

More recently, Holloway and Ansari [20] conducted a study to test the relationship between individual differences in primary school children’s NDE and achievement in math. In their study, 6–8 year-old children were required to compare numerical magnitudes ranging from 1–9 presented in a symbolic (Arabic digits) or nonsymbolic format (collection of black squares against a white background). The numerical distance between both nonsymbolic and symbolic numerical magnitudes ranged from 1 to 6. A significant negative relationship was found between math achievement and the size of the symbolic NDE; however, this relationship did not hold for the nonsymbolic NDE. These findings suggest that children who had larger symbolic NDE’s had poorer math skills. Given that developmental studies [15], [20] have shown that the NDE decreases over developmental time, the association between the magnitude of the NDE and arithmetic skills may suggest that children with relatively more immature (large) NDEs are also those that have comparatively poorer arithmetic abilities.

The work of Durand et al. [19] and Holloway and Ansari [20] each demonstrate a relationship between symbolic numerical magnitude processing and individual differences in children’s arithmetic skills; however, both of these studies were correlational in nature and used cross-sectional samples. The question remains whether individual differences in magnitude comparisons can *predict* individual differences in higher order math skills. To examine this matter, De Smedt, Verschaffel and Ghesquière [21] investigated whether numerical magnitude comparison has predictive value for individual differences in mathematical achievement. At the beginning of Grade 1 children completed a computerized symbolic numerical comparison task. Subsequently, at the beginning of Grade 2, children’s math achievement was assessed using a standardized achievement test for mathematics covering number knowledge, understanding operations, simple arithmetic, word problems and measurement. Results of their longitudinal study demonstrated that individual differences in children’s symbolic NDE, measured at the beginning of Grade 1, were related to achievement in math, as measured at the beginning of second grade. More specifically, children with small NDEs in Grade 1 tended to have higher scores on the standardized math assessment taken one year later. Furthermore, this association remained significant even when variables such as age, intellectual ability and speed of processing were controlled for.

Contrary to the findings by Holloway and Ansari [20] the relationship between numerical magnitude processing and achievement in math has also been demonstrated with nonsymbolic numerical magnitudes. In particular, Halberda, Mazzocco and Feigenson [22] investigated the relationship between individual differences in performance on a nonsymbolic number comparison task and variability in math achievement in a group of sixty-four 14 year-old children. These participants were followed longitudinally beginning from kindergarten to grade six and were annually given a large number of standardized measures of numerical and mathematical processing as well as standardized tests of IQ, vocabulary and working memory. In this study this group of children, at age fourteen, were shown an array of blue and yellow dots on a computer screen. These arrays were only presented for 200 ms making it too quick for participants to count. The accuracy of participants’ ability to compare numerical magnitudes was indexed using the Weber fraction. The Weber fraction provides a measure of the acuity with which an individual can discriminate between numerosities. As such, it is an indicator of the precision of one’s underlying mental representation of any numerical magnitude. Results demonstrated that individual differences in the Weber fraction not only correlated with individual differences in math achievement from kindergarten to grade six, but also retrospectively predicted math achievement of individual participants from as early as kindergarten. Furthermore, this relationship remained significant even when controlling for other potentially confounding cognitive variables such as working memory and reading. Findings from this study are significant in that they suggest that one’s acuity in comparing nonsymbolic magnitudes serves as a foundation for higher order math skills.

While Halberda, Mazzocco and Feigenson [22] demonstrated a relationship between nonsymbolic number comparison and math achievement in upper grades it raises the question whether this same relationship can be found in children before they receive formal instruction in math. More specifically, are individual differences in nonsymbolic magnitude comparison measured *before* formal schooling associated with later math performance? To follow this line of investigation, Mazzocco, Feigenson and Halberda [23] had 4 year-old children complete a nonsymbolic number comparison task in preschool and later assessed them at age 6 using standardized math tests. In their study children’s full scale IQ (FSIQ) and speed of processing were also assessed. The results of this study showed that individual differences in nonsymbolic magnitude comparison in preschool, as measured by the Weber Fraction, predicted math performance at age 6. In addition, these results also indicated that precision in this task at an early age was able to significantly predict later mathematical performance over and above other cognitive skills, again demonstrating the important role of numerical magnitude comparison ability for achievement in school mathematics.

In sum, while some studies suggest that symbolic but not nonsymbolic numerical magnitude comparison performance is related to children’s arithmetic skills, other studies have clearly shown that not only are nonsymbolic numerical magnitude processing skills correlated with children’s math performance but that such skills also predict arithmetic achievement over the course of developmental time. Few studies have conducted within-subject studies using both symbolic and nonsymbolic numerical magnitude processing and thus, it is unclear which of these might be a stronger, unique predictor of children’s arithmetic achievement scores.

Empirical findings such as those discussed above, raise the question whether or not a quick, efficient and classroom friendly assessment tool could be designed to formally measure basic magnitude processing in children. To partially address this question, Chard and colleagues [24] conducted a longitudinal study with kindergarten and Grade 1 students using a symbolic numerical comparison task. At the beginning of the school year (September), in the winter (January) and in the spring (May), participants were required to complete the task in which they were to verbally select the larger of two magnitudes ranging from 1–20. In the fall and spring of that same school year, they were also given the Number Knowledge Test [25] as a standardized assessment of math achievement. The Number Knowledge Test comprises a math assessment requiring participants to perform a variety of math skills such as counting, comparing magnitudes and completing simple arithmetic problems. Findings indicated that individual scores on the numerical comparison task correlated with children’s performance on the Number Knowledge Test at both test periods.

However, it is important to note that, similar to the aforementioned Durand et al. [19] study, Chard et al. [24] only examined symbolic magnitudes. Yet, as previously discussed, there is substantial evidence for an association between nonsymbolic magnitude processing and math abilities. Secondly, the Number Knowledge Test, like the number comparison task, requires individuals to compare numerical magnitudes. This weakens the correlational analysis conducted because the positive relationship revealed could, at least in part, reflect an association between two forms of number comparison. Finally, no other measures of cognitive performance were administered to participants. Without controlling for these cognitive processes it is impossible to know whether or not the relationship between magnitude comparison and math skills exists independently of other cognitive factors such as IQ, working memory and reading ability, all of which have been shown to correlate with children’s math achievement [26]–[29].

Taken together, previous research strongly suggests a relationship between, on the one hand, both symbolic and nonsymbolic number comparison and, on the other hand, individual differences in math achievement. Preliminary research has also demonstrated that an assessment of children’s symbolic magnitude processing is related to math performance, particularly arithmetic achievement [24]. What remains to be elucidated is whether a basic paper-and-pencil assessment, suitable for use in classrooms everywhere, measuring the accuracy of both children’s symbolic and nonsymbolic magnitude comparison abilities can reveal relationships between individual differences in numerical magnitude processing, both symbolic and nonsymbolic, and variability in arithmetic skills. Furthermore, whether a test of this kind can capture developmental changes in numerical magnitude processing also requires investigation. This is important because in order for results from such a test to be interpreted meaningfully, performance on the test should change as a function of chronological age (i.e., older children should perform better than younger children).

A basic paper-and-pencil assessment would be a valuable tool for several reasons. To begin, it would be very economical due to its low cost in comparison to computerized versions of the test that require specialized equipment and software. A test of this kind could also be quickly and easily administered and scored by the teacher in a large group setting. This would allow teachers to test the individual differences in basic numerical magnitude processing competence among their students. As this test would not require specialized software it could be used by educators in any setting such as schools with few resources or classrooms in developing countries and could be easily integrated into large scale studies that may be run by school boards, agencies or local governments.

The studies discussed above demonstrate that individual differences in basic magnitude processing are related to children’s math scores. In this context it is important to acknowledge that magnitude processing is not the only (or strongest) predictor of individual differences in math achievement. There is a large body of evidence demonstrating that math performance is related to cognitive abilities such as working memory. For example, working memory has been shown to play an important role in math skills such as solving both simple and complex arithmetic problems [26], [27]. Furthermore, poor working memory has been related to developmental disabilities in math [30]. Meanwhile, math performance has also been found to be related to literacy skills. For instance, Berg [28] and Koponen et al. [29] demonstrated a significant relationship between math achievement and reading. Similarly, De Smedt, Taylor, Archibald and Ansari [31] found a significant relationship between math performance such as arithmetic calculation and phonological processing. Thus, when studying the role played by basic numerical magnitude processing in math achievement, it is important to consider these other predictors and to estimate the unique variance explained by numerical magnitude processing measures.

In light of these findings, the objectives of the current study were threefold. First, we wanted to investigate whether a basic pencil-and-paper measure of symbolic and nonsymbolic number processing could characterize developmental changes in basic numerical magnitude processing, such as age-related improvement in accuracy of numerical comparisons. Our second goal was to explore whether performance on such a basic assessment tool of magnitude processing is capable of explaining variability in children’s math achievement scores and thirdly, we wanted to determine whether it explains significant variance over other factors such as working memory and reading skills.

## Methods

### Participants

A total of 197 students in Grades 1–3 participated in the current study. Eleven students were removed due to incorrect completion of the digit comparison task such as skipping pages of items or marking their responses in an unclear manner. Another four were removed from analysis due to performing at ceiling on the task (that is, they completed all trials correctly within the time-limit allotted). Twelve more children were removed due to their inability to reach a basal score on the Math Fluency and Calculation subtests of the Woodcock-Johnson III Subtests of Achievement (WJ III; see below). For the Math Fluency test, any participant who had three or fewer items correct after one minute did not reach basal. For the Calculation test, if a child did not respond correctly to at least one of two practice items, the child did not reach basal and testing was discontinued. Five children were not able to reach basal on the Reading Fluency test of the WJ III; that is, they had fewer than three items correct on the four practice exercises. Three children did not reach basal on the Vocabulary subtest of the Wechsler Abbreviated Scale of Intelligence **(**WASI; see below). In the Vocabulary subtest of the WASI, testing began on the fourth item. If the participant did not receive a perfect score on the fourth and fifth items, then the examiner administered the first three items in reverse order. Testing was discontinued after three consecutive scores of zero. In the Automated Working Memory Assessment (AWMA; see below), one child did not reach basal on the Spatial Recall subtest and one child did not reach basal on the Listening Recall subtest meaning the participant failed to correctly answer the first three items on each subtest. For each subtest of the AWMA, testing was discontinued if the participant failed to correctly answer the first three items. Therefore, our final sample included 160 children (83 females) between the ages of 6 years, 4 months and 9 years, 7 months (M = 8 years, 1 month, SD = 9.38 months). Twenty-six children were in Grade 1 (M = 6 years; 8 months, SD = 3.71 months), 56 children were in Grade 2 (M = 7 years; 8 months, SD = 3.43 months) and 78 children were in Grade 3 (M = 8 years; 8 months, SD = 3.43 months). All participants spoke English fluently and had normal or corrected to normal vision.

Permission was granted from a local school board and school principals to recruit students from elementary schools in a region of Southwestern Ontario. Letters of information and consent forms approved by the University of Western Ontario’s Research Ethics Board were received and completed by parents of the participants before the study began. Interested parents representing 36 schools in both urban and rural areas consented to having their child(ren) participate in the current study. Participants were from various socioeconomic and ethnic groups.

### Tests and Materials

#### Magnitude comparison

During the magnitude comparison task participants were required to compare pairs of magnitudes ranging from 1–9. Stimuli were given in both symbolic (56 digit pairs) and nonsymbolic (56 pairs of dot arrays) formats. In both formats of presentation, each numerical magnitude was counterbalanced for the side of presentation (i.e., 2|7, 7|2). Furthermore, in the nonsymbolic form, dot stimuli were controlled for area and density.

To control for area and density, half of the dot arrays used were matched for total area and half of the dot arrays were matched for total perimeter. In other words, half of the trials had equal area while the other half had equal perimeter. The array with the most dots had a greater perimeter when cumulative surface area was matched. The array with the most dots had more cumulative surface area when perimeter was matched. To avoid having the participant rely on the relative size of the dot arrays, both perimeter-matched and area-matched trials were presented randomly. To ensure that the test items became increasingly more difficult, the numerical ratio between the numerical magnitudes presented was manipulated. Easier items (with smaller ratios) were presented first and more difficult items were presented next (increasingly larger ratios). By starting with the easier items, this ensured that children remained motivated to complete the task. The order of trials in our assessment was similar to the order of ratios presented in Table 1. Order was slightly varied between symbolic and nonsymbolic conditions to ensure that the order of presentation of items was not identical between conditions, but both followed a similar pattern where pairs of symbolic and nonsymbolic stimuli with relatively smaller ratios were presented before larger ratios. The ratio (small/large) between numerical pairs ranged from.11 to.89, for example the ratio between 3 and 5 is.60 (see Table 1 for pairs and ratios used).

**Table 1:** Numerical pairs and ratios for the numerical comparison task.

Number pair	Ratio
1–9	.11
1–8	.13
1–7	.14
1–6	.17
1–5	.20
2–9	.22
2–8	.25
2–7	.29
3–9	.33
3–8	.38
2–5	.40
3–7	.43
4–9	.44
3–6	.50
4–8	.50
5–9	.56
4–7	.57
3–5	.60
5–8	.63
2–3	.67
5–7	.71
6–8	.75
7–9	.78
4–5	.80
5–6	.83
6–7	.86
7–8	.88
8–9	.89

**Figure 1:**
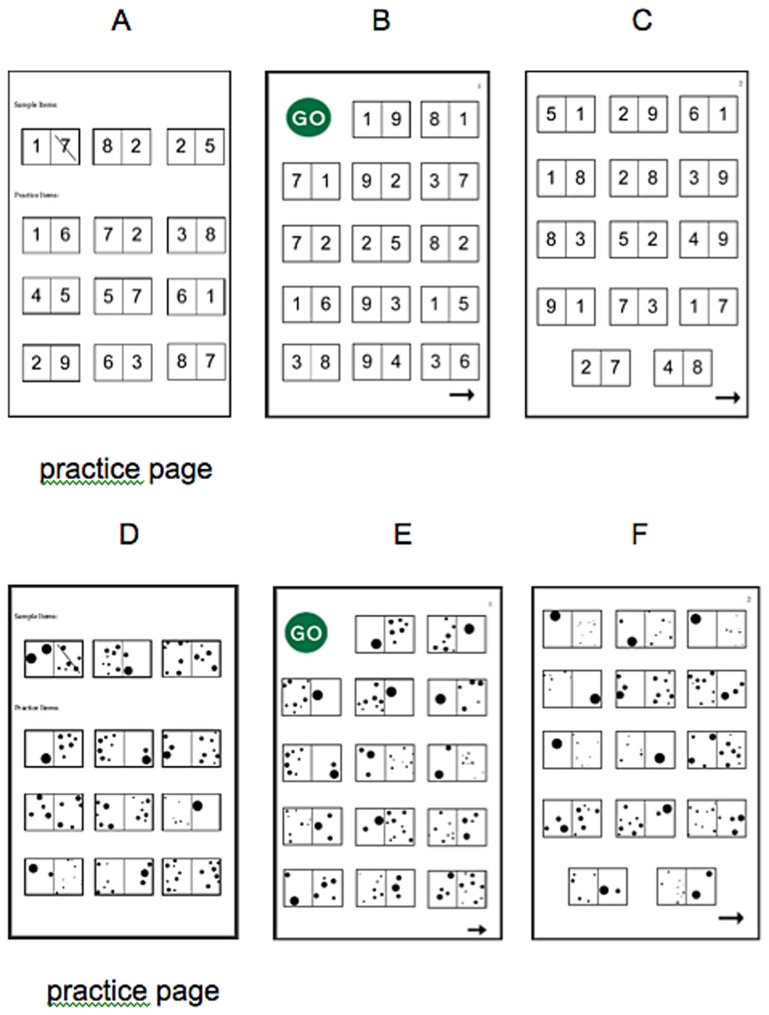
Paper-and-pencil measure. Figures A, B, and C are examples of symbolic items. Figures D, E and F are examples of nonsymbolic items.

During the test, participants were told to cross out the larger of the two magnitudes and were given one minute to complete the symbolic condition and one minute to complete the nonsymbolic condition. To ensure that participants understood the task, each child completed three sample items with the examiner and then nine practice items on their own before beginning the assessment (see figs. 1a & 1d). This was done for both symbolic and nonsymbolic conditions. During the instructions given for the nonsymbolic condition, participants were told not to count the dots. Examiners were again able to emphasize this instruction during the participants’ completion of the practice items. The order of format presentation was varied in such a way that half of the students in each grade received the symbolic items first and the other half received the symbolic items second (see fig. 1 for sample of test pages).

#### Arithmetic skills

In order to determine the subjects’ competence in mathematics, the Woodcock-Johnson III Subtests of Achievement (WJ III [32]) was used. Each child was required to complete the Math Fluency and Calculation subtests. The Calculation subtest measures skills in mathematical computations. The individual is required to perform addition, subtraction, multiplication and division and combinations of these operations. There is no time constraint. The Math Fluency test assesses one’s ability to quickly solve simple arithmetic problems. The participant is given three minutes to complete as many addition, subtraction and multiplication problems as possible. It should be noted that neither of the subtests contained any item that required numerical comparison.

#### Reading skills

In order to assess the reading ability of each participant, children were given the Reading Fluency subtest of the WJ III [32]. This test requires the individual to quickly read simple sentences and to decide if the sentences are true or false by circling “yes” or “no” in the response booklet.

#### Intelligence

Cognitive performance was measured using two subtests of the Wechsler Abbreviated Scale of Intelligence (WASI [33]).

*Vocabulary:* Items of the Vocabulary subtest assess the individual’s ability to define words. Initial items require subjects to name pictures of objects. Later items require subjects to verbally define words that are read by the examiner.

*Block Design:* During this subtest the child is given a specific time frame to manipulate blocks with the goal of replicating a stimulus design that has been visually presented.

#### Working memory

The Automated Working Memory Assessment (AWMA [34]) is a standardized computer-based tool used to assess both verbal and visual-spatial working memory skills. Verbal working memory was measured using the Counting Recall and Listening Recall sub-tests while visual-spatial working memory was measured using the Odd-One-Out and Spatial Recall sub-tests. All tasks follow a span procedure such that items in the list increase when the child completes at least 4 of 6 lists correctly and the task is discontinued when the child fails three items at any list length.

*Counting Recall:* During this task, students count the circles in a series of shape arrays and are required to recall the serial totals verbally. At each level the task becomes increasingly difficult as the number of arrays shown increases.

*Listening Recal:* This task requires the individual to listen to a sentence, to decide if the statement is true or false and then to repeat the last word of the phrase heard. As the test continues, participants are presented with two to a maximum of six sentences at a time.

*Odd-One-Out:* During this subtest, the child is quickly presented with three stimuli of which one is slightly different than the others. The child is required to point to the “odd-one-out” and is then presented with another screen on which the stimuli are replaced by three blank squares. The child is then asked to point to where the stimulus that was the odd-one-out was originally located. In subsequent trials, the subject is presented with up to seven different sets of stimuli in a row after which he or she is presented with the screen with the blank squares and is asked to point to where each odd stimulus was located in the same order in which they were originally presented.

*Spatial Recall:* During this task, individuals are shown two stimuli on a computer screen that are either oriented in a similar direction or in an opposite fashion. The stimulus on the right also has a red dot located at one of three positions. The participant is first required to determine whether the stimuli are oriented in a similar or opposite fashion by saying “same” or “opposite”. Following this, another screen is presented which displays three black dots corresponding to the three possible positions for the red dots presented with stimuli on the right from the previous screen. In this case the child is asked to point to one of the black dots to indicate where the red dot had been located on the original stimuli.

#### Procedure

The current study was part of a large-scale study wherein children’s reading, math and language skills were tested. All participants were assessed at their respective elementary school in three one-hour sessions over a period of three weeks at the end of the school year. Each participant was tested individually by trained examiners in a quiet area outside of the classroom.

## Results

**Figure 2:**
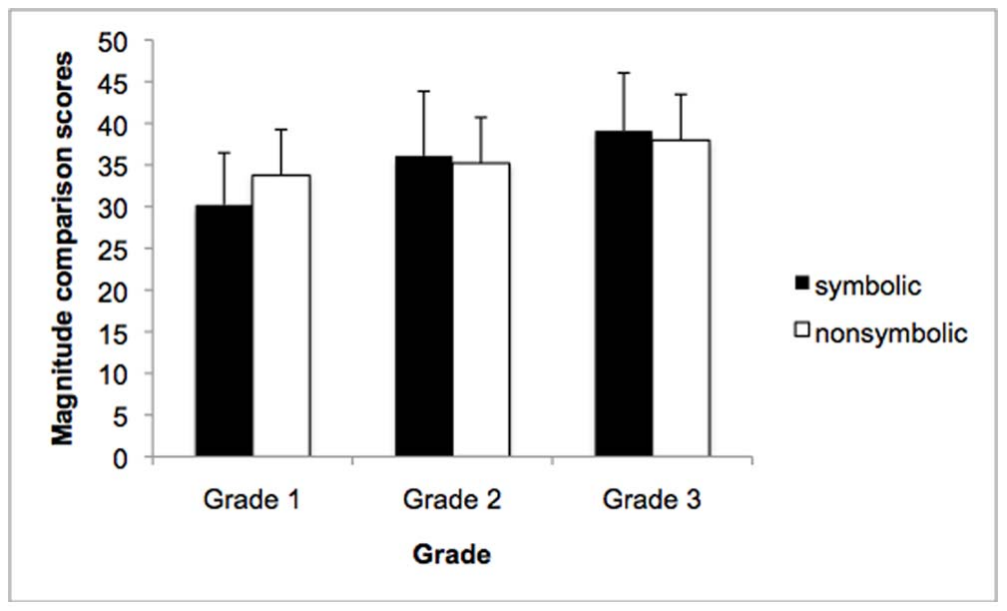
Grade by format interaction. Bar graph representing overall performance of participants in each grade for symbolic and nonsymbolic items. Grade 1 participants were significantly better at nonsymbolic items compared to symbolic items. Participants in grades 2 and 3 did not demonstrate any differences between conditions. Standard errors are represented by the error bars attached to each column.

The means and standard deviations for the tests used are shown in Table 2. In order to identify whether this assessment could identify age-related differences in magnitude processing, a repeated measures ANOVA using format (symbolic and nonsymbolic) as a within subjects variable and grade (1^st^, 2^nd^ and 3^rd^ grades) as a between subjects variable was conducted. Analyses revealed no main effect of format (*F*(1, 157) = .311, *ns*), a main effect of grade (*F*(2, 157) = 14.18, *p*<.001, *η* ^2^ = .15 ) and a format×grade interaction, (*F*(2, 157) = 6.61, *p*<.001, *η* ^2^ = .08; Fig. 2), whereby Grade 1 children were more accurate on the nonsymbolic items (*t*(25) = −3.21, *p*<.05) compared to symbolic items. In contrast, there was no significant difference between formats in the Grade 2 (*t*(55) = 1.38, *p = *.17) or Grade 3 (*t*(77) = 1.40, *p* = .165) participants.

**Table 2:** Means and Standard Deviations (S.D.).

Test	N	Mean Raw scores (S.D.)	Range (min.-max.)	Mean standard scores (S.D.)	Range (min.-max.)
Age (months)	160	97.54 (9.38)	77–115	N/A	N/A
Symbolic	160	36.65 (7.82)	16–55	N/A	N/A
Nonsymbolic	160	36.40 (6.01)	21–54	N/A	N/A
Math Fluency	160	31.23 (13.05)	4–75	92.60 (13.60)	65–136
Calculation	160	10.26 (3.09)	1–17	95.05 (15.36)	29–135
Listening Recall	160	10.00 (3.04)	4–20	103.29 (11.45)	78–135
Counting Recall	160	15.56 (4.35)	5–31	103.31 (13.74)	71–133
Odd-One-Out	160	17.50 (4.14)	3–29	110.76 (13.24)	71–133
Spatial Recall	160	14.35 (4.68)	1–26	104.84 (13.61)	69–137
Vocabulary^1^	160	28.04 (5.86)	13–43	49.73 (8.49)	29–69
Block Design^1^	160	16.51 (10.11)	3–48	53.65 (10.14)	34–80
Reading Fluency	160	28.66 (11.37)	2–57	101.90 (10.51)	75–142

*Note.* Symbolic - total correct scores on symbolic items; Nonsymbolic - total correct scores on nonsymbolic items; Math Fluency –scores received on WJ-III; Calculation – scores received on WJ-III; Listening Recall – scores received on AWMA; Counting Recall – scores received on AWMA; Odd-One-Out – scores received on AWMA; Spatial Recall – scores received on AWMA; Vocabulary – scores received on WASI; Block Design – scores received on WASI; Reading Fluency – scores received on WJ-III.

1 The WASI uses a population mean of 50 and standard deviation of 10.

### Correlations

Correlations were calculated for the following variables across all three grades (see Table 3): Math Fluency raw scores, Calculation raw scores, verbal working memory raw scores, visual-spatial working memory raw scores, symbolic score (total number of correctly solved symbolic comparison trials), nonsymbolic score (total number of correctly solved nonsymbolic comparison trials), total score (total number of correctly solved comparison trials across both symbolic and nonsymbolic), IQ raw scores and Reading Fluency raw scores. To perform this analysis, a partial correlation was performed controlling for age. In other words, the effect of chronological age on participants’ raw scores on all standardized tests was removed. We chose to use raw scores in our analysis, because in a preliminary analysis it was found that age negatively correlated with Math Fluency, Calculation, IQ and Reading Fluency standard scores. Such a negative correlation is not expected because standard scores are adjusted for chronological age and thus there should be no relationship between chronological age and standard scores. By using the raw scores, we are not using a measurement that is related to a reference group that may not be fully representative of the one tested in the present study.

**Table 3:** Partial correlations controlling for age in months (Gr. 1–3).

Variable	1	2	3	4	5	6	7	8	9	10	11	12
1. MF	–	.64**	.40**	.45**	.38**	.28**	.34**	.30**	.17*	.43**	.33**	.43**
2. MC		–	.31**	.35**	.28**	.29**	.43**	.41**	.35**	.35**	.26*	.34**
3. RF			–	.32**	.13	.39**	.19*	.33**	.05	.31**	.27*	.33**
4. OOO				–	.51**	.31**	.40**	.22*	.27*	.31**	.15	.26*
5. SR					–	.22*	.26*	.15	.30**	.21*	.12	.19*
6. LR						–	.44**	.32**	.05	.18*	.12	.18*
7. CR							–	.33**	.23*	.15	.03	.11
8. Vocab								–	.25*	.16*	.11	.16*
9. BD									–	.20*	.34**	.30**
10. Sym										–	.59**	.92**
11. Nonsym											–	.87**
12. Overall												–

*Note.* MC - Calculation; MF - Math Fluency; RF - Reading Fluency; OOO – Odd-one-out; SR – spatial recall; LR– Listening recall; CR – Counting recall; Vocab – vocabulary; BD – Block design; Sym – symbolic mean score; Non-sym – nonsymbolic mean score; Overall – overall mean score.

* *p*<.05.

** *p<*.01.

**Figure 3:**
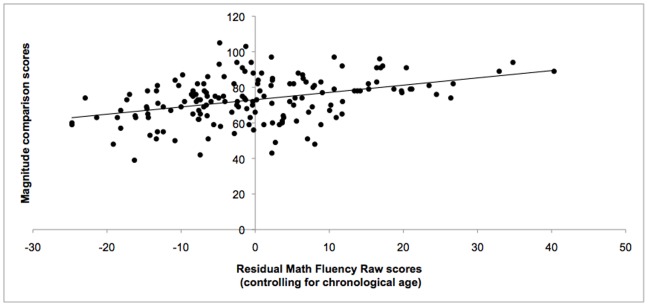
Correlation between Math Fluency scores and magnitude comparison scores. Scatterplot showing significant correlation between standard scores on the Math Fluency subtest of the Woodcock-Johnson III battery and overall mean score of the magnitude comparison task (symbolic and nonsymbolic combined) for all participants. The solid line represents the linear regression line for this relationship.

**Figure 4:**
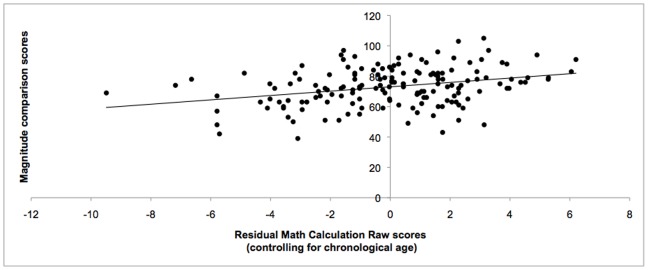
Correlation between Calculation scores and magnitude comparison scores. Scatterplot showing significant correlation between standard scores on the Calculation subtest of the Woodcock-Johnson III battery and overall mean score of the magnitude comparison task (symbolic and nonsymbolic combined) for all participants. The solid line represents the linear regression line for this relationship.

As seen from Table 3, the total score (symbolic and nonsymbolic combined) on the magnitude comparison task significantly correlated with Math Fluency and Calculation scores (see Figs. 3 & 4). The total score also correlated with each IQ subtest and each working memory subtest except Counting Recall. Symbolic and nonsymbolic scores each significantly correlated with Math Fluency, Calculation, and Reading Fluency. Symbolic mean scores were found to significantly correlate with each standardized test with the exception of Counting Recall. Nonsymbolic test scores correlated with the Block Design subtest, but did not significantly correlate with the Vocabulary subtest, nor any of the working memory subtests. Both Math Fluency and Calculation correlated significantly with each of the standard tests that were administered. Reading Fluency correlated with all measures except Spatial Recall and Block Design. Turning to memory skills, Odd-One-Out scores correlated with each standardized measure. Spatial Recall correlated with each standardized assessment with the exception of Vocabulary. Listening Recall correlated with each standardized assessment except Vocabulary and Counting Recall scores correlated with all measures except Block Design.

Further analyses were conducted on the significant association between magnitude comparison and arithmetic achievement to examine the relationship between performance on the paper-and-pencil assessment and test scores for each grade level. As can be seen in Table 4, for Grade 1, we found no significant relationship between Math Fluency scores and performance on the symbolic items (*r* = .34, *ns*) neither between Math Fluency scores and nonsymbolic items (*r* = .25, *ns*). There was, however, a significant relationship between Calculation scores and symbolic performance (*r* = .52, *p*<.01), however there was no correlation between Calculation scores and performance on nonsymbolic items (*r* = .25, *ns*). Table 5 demonstrates that in Grade 2, a significant relationship between students’ Math Fluency scores and symbolic performance (*r* = .42, *p*<.01) and also between Math Fluency scores and nonsymbolic performance (*r* = .33, *p*<.05) was obtained. In addition, there was also a significant relationship between Calculation performance and symbolic scores (*r* = .31, *p*<.01), but there was no significant correlation between Calculation and nonsymbolic performance (*r* = .15, ns). Participants in the third grade (see Table 6) demonstrated a significant relationship between Math Fluency scores and symbolic items (*r* = .45, *p*<.01) as well as a significant correlation between Math Fluency and nonsymbolic items (*r* = .33, *p*<.01). Significant associations were also found between Calculation scores and symbolic scores (*r* = .30, *p*<.01) along with a significant correlation between Calculation scores and nonsymbolic performance (*r* = .35, *p*<.01).

**Table 4:** Grade 1 correlations between arithmetic achievement and magnitude comparison.

Variable	1	2	3	4	5
1. MF	–	.73**	.34	.25	.34
2. MC		–	.52**	.25	.44*
3. Sym			–	.56**	.88**
4. Nonsym				–	.87**
5. Overall					–

*Note.* MC – Calculation raw scores; MF - Math Fluency raw scores; Sym – symbolic mean.

score; Nonsym – nonsymbolic mean score; Overall – overall mean score.

* *p*<.05.

** *p<*.01.

**Table 5:** Grade 2 correlations between arithmetic achievement and magnitude comparison.

Variable	1	2	3	4	5
1. MF	–	.59**	.42**	.33*	.41**
2. MC		–	.31*	.15	.27*
3. Sym			–	.68**	.94**
4. Nonsym				–	.88**
5. Overall					–

*Note.* MC – Calculation raw scores; MF - Math Fluency raw scores; Sym – symbolic mean score; Nonsym – nonsymbolic mean score; Overall – overall mean score.

* *p*<.05.

** *p<*.01.

**Table 6:** Grade 3 correlations between arithmetic achievement and magnitude comparison.

Variable	1	2	3	4	5
1. MF	–	.62**	.45**	.33**	.45**
2. MC		–	.30**	.35**	.37*
3. Sym			–	.56**	.90**
4. Nonsym				–	.86**
5. Overall					–

*Note.* MC – Calculation raw scores; MF - Math Fluency raw scores; Sym – symbolic mean score; Nonsym – nonsymbolic mean score; Overall – overall mean score.

* *p*<.05.

** *p<*.01.

We then examined whether this grade-related difference in the strength of the correlations between, on the one hand, the symbolic and nonsymbolic performance and, on the other hand, Math Fluency and Calculation scores were statistically significant. In other words, whether the nonsignificant correlations in Grade 1 differed significantly from the significant correlations in the other grades. To do this we transformed correlation coefficients into Fisher’s *z* statistics and then made comparisons using a *z* test. For the association between the symbolic items and Math Fluency scores, the correlation for the Grade 1 students was not significantly different from that of the Grade 2 students (*z* = −0.37, *ns*) or the Grade 3 students (*z* = −0.55, *ns*). The difference between the Grade 2 and Grade 3 correlations was also not significant (*z* = −0.21, *ns*). Similarly, for the association between the nonsymbolic items and Math Fluency scores, the correlation between the students in Grade 1 compared to the correlation for Grade 2 students was not significantly different (*z* = −0.35, *ns*) or for the students in the third grade (*z* = −0.37, *ns*). The difference between the correlations for Grade 2 and Grade 3 were also nonsignificant (*z* = −0.03, *ns*). Likewise, for the relationship between performance on symbolic items and Calculation scores, the correlation coefficient for Grade 1 was once more not significantly different from the correlation for either Grade 2 (*z* = 1.02, *ns*) or for Grade 3 (*z* = 1.12, *ns*). Additionally, the correlation for the Grade 2 students did not differ significantly from the correlation for students in Grade 3 (*z* = .006, *ns*). Finally, the differences found between the correlations of nonsymbolic items and Calculation scores were nonsignificant between the Grade 1 and Grade 2 students (*z* = 0.42, *ns*) as well as the Grade 1 and Grade 3 students (*z* = −.046, *ns*). Similarly, no significant difference was found between the correlations of the Grade 2 and Grade 3 students (*z* = −1.19, *ns*).

Thus while the correlations in Grade 1 between math scores and symbolic and nonsymbolic performance on the paper-and-pencil test do not pass the threshold for statistical significance (likely due to the comparatively small sample size), these correlations do not significantly differ from the ones in grades two and three. Therefore, a true developmental change in the relationships between arithmetic performance and the present measure of symbolic and nonsymbolic numerical magnitude processing cannot be supported by the present data. Instead the difference in the correlational strengths is likely due to differential sample sizes and, importantly, the correlations are significant when all three samples are collapsed into on group.

### Regression

Since Reading Fluency, verbal working memory, visual-spatial working memory and IQ each correlated with children’s scores on Math Fluency and Calculation, the specificity of the key relationship between number comparison and arithmetic skills needed to be further investigated. To do so, two linear regressions were performed: one to examine the relationship between Math Fluency (dependent variable), symbolic and nonsymbolic total score while controlling for age, verbal working memory, visual-spatial working memory, IQ and Reading Fluency; and the other, to examine the relationship between Calculation (dependent variable), symbolic and nonsymbolic total score while controlling for age, verbal working memory, visual-spatial working memory, IQ and Reading Fluency. Since no hypotheses were made about the order of predictors and, in an effort to investigate which variables accounted for significant unique variance, all predictor variables were entered as one step (see Tables 7 & 8).

**Table 7:** Linear regression analyses predicting Math Fluency raw scores with chronological age, Reading Fluency, visual spatial working memory, verbal working memory, IQ, symbolic scores and nonsymbolic scores as predictors.

Math Fluency		
Predictor	β	*t*
Age	.014	.187
Reading	.208*	2.49
Odd-One-Out	.148	1.91
Spatial Recall	.183*	2.51
Listening Recall	−.029	−.375
Counting Recall	.159*	2.14
Vocabulary	.088	1.24
Block Design	−.066	−.912
Symbolic	.197*	2.35
Nonsymbolic	.128	1.56

* *p<*.05.

**Table 8:** Linear regression analyses predicting Calculation raw scores with chronological age, Reading Fluency, visual spatial working memory, verbal working memory, IQ, symbolic scores and nonsymbolic scores as predictors.

Calculation		
Predictor	β	*t*
Age	.126	1.72
Reading	.126	1.53
Odd-One-Out	.027	.355
Spatial Recall	.049	.693
Listening Recall	.020	.268
Counting Recall	.226*	3.11
Vocabulary	.157*	2.26
Block Design	.186*	2.61
Symbolic	.170*	2.07
Nonsymbolic	.013	.164

* *p<*.05.

Results demonstrated that our first linear regression using Math Fluency as a dependent variable was significant (*F*(10, 159) = 14.41, *p*<.001, *R* ^2^ = .492). In this model we found that only performance on Reading Fluency, Spatial Recall, Counting Recall and symbolic items account for significant unique variance in Math Fluency. Performance on nonsymbolic items did not account for significant unique variance in Math Fluency.

The second regression analysis using Calculation as a dependent variable was also significant (*F*(10, 159) = 15.67, *p*<.001, *R^2^* = .513) and demonstrated that performance on Counting Recall, Vocabulary, Block Design and symbolic items account for significant unique variance in Calculation. Again, as in Math Fluency, performance on nonsymbolic items did not account for significant unique variance.

## Discussion

The purpose of this study was to extend previous research in three principal ways: 1) to investigate whether a basic paper-and-pencil measure of symbolic and nonsymbolic numerical magnitude processing could be used to measure age-related changes in basic numerical magnitude processing skills, 2) to explore whether performance on this basic assessment tool is related to individual differences in children’s performance on measures of arithmetic achievement, and 3) to determine whether it explains significant variance over other factors such as age, working memory, reading skills and IQ.

With regards to the first aim of our study, we found age-related differences in the performance of children on the paper-and-pencil measure. Specifically, analyses demonstrated a main effect of grade, which indicates that children improved in the magnitude comparison task as they became older, replicating previous findings and suggesting that this test, like computerized measures, can be used to characterize developmental changes in numerical magnitude processing. Furthermore, a format by grade interaction was also found whereby Grade 1 students were the only age group that performed significantly better on the nonsymbolic than symbolic items. This finding demonstrates that younger children were more accurate at nonsymbolic number processing than symbolic processing, whereas older children did not show this difference. These results indicate that over the course of developmental time, typically developing children become more proficient with symbolic number processing as they progress in school and acquire more familiarity and automaticity with numerical symbols. Moreover, it also suggests that perhaps young children have strong pre-existing representations of nonsymbolic numerical magnitude (that can even be found in infancy) and only gradually map these onto symbolic representations.

The results from the current study also demonstrated that participants’ scores on this basic assessment tool significantly correlated with their scores on standardized tests of arithmetic achievement. More specifically, a significant positive relationship was found between Math Fluency, Calculation and the accuracy with which participants completed the symbolic items, nonsymbolic items and overall total scores on the magnitude comparison task. This finding indicates that children who scored highly on Calculation and Math Fluency also tended to receive high scores on our test. This association of numerical magnitude comparison skills and individual differences in arithmetic skills replicates findings in earlier work. For instance, the positive correlation found in the current study between performance on a timed numerical comparison task and individual differences in arithmetic performance replicates the work of Durand, Hulme, Larkin and Snowling [19], but provides further constraints not afforded by prior research. For example, Durand, Hulme, Larkin and Snowling [19] only used digits from 3–9 with digit pairs differing only by a magnitude of 1 or 2. By including a larger range of digits, greater magnitudes separating each digit pair, as well as nonsymbolic stimuli in the current study, our results significantly expand upon Durand et al.’s [19] findings. For example, including nonsymbolic items could allow for this test to be used with children who do not yet have an understanding of number symbols.

Finally, a key finding from our study indicated that performance on the symbolic items accounts for unique variance in arithmetic skills. Interestingly, this same result was not found for performance on the nonsymbolic items as demonstrated in previous research [22], [23].

Specifically, we found that while simple correlations show that both are related to arithmetic achievement, when we examined which of them accounts for unique variance, using multiple regression analyses, only symbolic magnitude comparison was found to account for unique, significant variance in children’s performance on the standardized tests of arithmetic achievement. Since the simple correlations revealed that accuracy on both the symbolic and nonsymbolic tasks independently correlated with math achievement, it is possible that they share variance related to core magnitude processing, but that nonsymbolic does not contribute any additional, unique variance to math performance while symbolic does. We speculate that the unique variance accounted for by symbolic processing is related to recognizing numerals and mapping numerals to magnitudes – a skill that is important in the mental manipulation of digits during calculation. While it is possible that symbolic and nonsymbolic share variance related to numerical magnitude processing, it is equally plausible that their shared variance (and the absence of unique variance accounted for by the nonsymbolic task) is explained by non-numerical factors that are tapped by both tasks, such as speed of processing, attention, working memory or a complex combination of these factors and numerical magnitude processing. It is impossible to arbitrate between these different explanations given the current data. However, what the current data show are that symbolic number comparison explains unique variance while nonsymbolic does not, strengthening the notion that the mapping of symbols to numerical magnitudes is a critical correlate of individual differences in children’s arithmetic achievement [20], [35], [36].

While children’s performance on the symbolic items of our test accounts for unique variance in arithmetic performance it is not the greatest predictor of arithmetic achievement. For example, the counting recall task of the AWMA accounted for variance in Calculation performance over and above symbolic number comparison scores. This demonstrates that while our test does account for some unique variability in children’s arithmetic skills, other number related abilities as well as measures of working memory, such as the counting recall task, also play an important role in children’s arithmetic skills. This should be considered and investigated further in future research of this kind.

Finally, the results from the multiple regression reveal, as previous studies have demonstrated [26], [27] that measures of both verbal and non-verbal working memory account for unique variance in children’s arithmetic scores. What is novel about the present finding is that both working memory and symbolic number processing skills account for unique variance, suggesting that these competencies are not confounded with one another in predicting individual differences in children’s arithmetic skills.

The age range of our sample and measures of math achievement used in the current study are very similar to the work done by Holloway and Ansari [20]. Using a computerized paradigm of symbolic and nonsymbolic magnitude comparison, Holloway and Ansari [20] investigated the relationship between basic magnitude processing skills in 6–8 year-old children and arithmetic abilities using the same standardized tests of math achievement as the current study. They found that participants’ performance on symbolic, but not nonsymbolic magnitude comparison significantly correlated with math achievement scores. Interestingly, these correlations were strongest for the 6-year old children and weaker and nonsignificant, in older age groups (7 and 8 years) tested, which suggested a developmental trend. However, as detailed in the paper by Holloway and Ansari [20] further analyses revealed that there was no significant difference between the correlations for symbolic performance and test scores between the different age groups. Therefore, in the absence of significant differences between correlation coefficients they were unable to make any developmental claims.

Our findings also suggested a developmental trend whereby the relationship between symbolic performance and math achievement became stronger and more significant the older the participants, which may be construed to be contrary to the findings reported by Holloway and Ansari [20]. However, like Holloway and Ansari [20] we also did not find any significant difference in the relationship between the correlations for symbolic performance and math achievement at each grade level. Again, since there is no evidence of significant differences between correlation coefficients we are also unable to make any claims regarding developmental trends. Therefore, direct conclusions about the differences between developmental trajectories in both papers cannot be made, since in neither paper differences in the strength of correlations between age groups/grades were found to be significant. Importantly, both our results and those reported by Holloway and Ansari [20] demonstrates that when controlling for chronological age, the performance of children between the ages 6–9 on measures of symbolic numerical magnitude comparison significantly correlate with between-subjects variability on standardized measures of arithmetic achievement. In this way there is convergence between the results reported by Holloway and Ansari [20] and those detailed in this report.

As seen in Table 2, there is a large difference between, on the one hand, Math Fluency and Calculation scores and, on the other hand, Reading Fluency scores in our sample. However, though the Math Fluency and Calculation scores are below average they are still within the normal range (85–115). Moreover, in other studies we have conducted with children in our local school district we have found similar average results. Thus the scores from our present sample are convergent with what we are finding in our local area more generally. This may therefore be a consequence of the current educational policy in the province of Ontario, which places a stronger emphasis on problem solving over fluency in math. Consequently, our sample is a little discrepant from the standardization sample. However, in our current analysis we use raw scores and thus do not rely on standardized results. Furthermore, while the average for math scores is lower than 100 there is large variability in the scores with children performing both above and below the normal range. Thus, we believe that while we have a sample with an average below 100 (though still in the normal range) this large variability in math scores found in our sample allows us to meaningfully capture individual differences.

Unfortunately, there were a greater number of parents of children in grades two and three who agreed to have their children participate in the study than parents of children in Grade 1. These practical constraints of the study led to considerable differences in sample size between grade levels. Future investigations of this kind should therefore be conducted using equal sample sizes.

In sum, the current results demonstrate that a relationship exists between performance on a basic magnitude comparison task and individual differences in math achievement (as measured by arithmetic skills). Furthermore, it was found that symbolic processing accounts for unique variance in arithmetic skills while nonsymbolic processing does not. Finally, results indicate that a measure of this kind can characterize developmental changes in basic numerical magnitude processing.

As mentioned, previous research has shown that children who have strong skills in higher order mathematics, such as arithmetic, also demonstrate strong magnitude processing skills. The measurement tool investigated in the current study will allow educators the opportunity to quickly and easily assess these foundational competencies. A test of this kind will also help educators to focus on these essential skills during math instruction in the classroom. By focusing on these basic, yet foundational abilities educators can directly foster the numerical magnitude processing abilities of their students.

In addition, previous research has shown that not all measures of basic number processing correlate with individual differences in math achievement [37]. Therefore, a differentiated understanding of basic number processing and its relationship to arithmetic achievement is needed. In this regard, future studies should investigate the relationship between our assessment and other measures of magnitude processing such as response time measures, Weber fractions and number line estimation tasks.

In the current study, we found that children’s performance on nonsymbolic items correlated with their arithmetic skills. This may suggest that the nonsymbolic portion of our assessment may be used by itself with preschool children and children that do not yet have a semantic representation of number symbols, further demonstrating the utility of this simple assessment. Future studies would have to be used to investigate this line of research. In addition, future research should seek to examine the reliability of the number comparison assessment by measuring the test-retest reliability of this assessment tool. Using a longitudinal design, forthcoming research should also seek to investigate this assessment tool and its predictive ability to identify children who are at risk for developing difficulties in mathematics. Such research is critical, as the current findings are merely correlational and may indicate that basic magnitude processing facilitates math development, but performance on the test may equally well reflect the fact that greater practice with arithmetic leads to improved performance in numerical magnitude comparison. A test that has the potential to truly predict individual differences in arithmetic ability would be a significant contribution to scores of classrooms and could have a great impact on the future of many students. By identifying at-risk children earlier and more reliably, findings from this and future studies will put us one step closer to improving the numeracy skills of students with difficulties in math and possibly enhance the teaching strategies currently used to instruct this specific group of children.
